# Bioorthogonally surface‐edited extracellular vesicles based on metabolic glycoengineering for CD44‐mediated targeting of inflammatory diseases

**DOI:** 10.1002/jev2.12077

**Published:** 2021-03-12

**Authors:** Gyeong Taek Lim, Dong Gil You, Hwa Seung Han, Hansang Lee, Sol Shin, Byeong Hoon Oh, E. K. Pramod Kumar, Wooram Um, Chan Ho Kim, Seungsu Han, Sangho Lee, Seungho Lim, Hong Yeol Yoon, Kwangmeyung Kim, Ick Chan Kwon, Dong‐Gyu Jo, Yong Woo Cho, Jae Hyung Park

**Affiliations:** ^1^ School of Chemical Engineering Sungkyunkwan University Suwon Republic of Korea; ^2^ Department of Health Sciences and Technology SAIHST Sungkyunkwan University Suwon Republic of Korea; ^3^ Department of Biological Sciences Sungkyunkwan University Suwon Republic of Korea; ^4^ Center for Theragnosis Biomedical Research Institute Korea Institute of Science and Technology Seoul Republic of Korea; ^5^ School of Pharmacy Sungkyunkwan University Suwon Republic of Korea; ^6^ ExoStemTech Inc. Ansan Republic of Korea; ^7^ Department of Chemical Engineering Hanyang University Ansan Republic of Korea

**Keywords:** biodistribution, bioorthogonal copper‐free click chemistry, CD44‐targeting, extracellular vesicles, metabolic glycoengineering

## Abstract

Extracellular vesicles (EVs) are essential mediators in intercellular communication that have emerged as natural therapeutic nanomedicines for the treatment of intractable diseases. Their therapeutic applications, however, have been limited by unpredictable in vivo biodistribution after systemic administration. To control the in vivo fate of EVs, their surfaces should be properly edited, depending on the target site of action. Herein, based on bioorthogonal copper‐free click chemistry (BCC), surface‐edited EVs were prepared by using metabolically glycoengineered cells. First, the exogenous azide group was generated on the cellular surface through metabolic glycoengineering (MGE) using the precursor. Next, PEGylated hyaluronic acid, capable of binding specifically to the CD44‐expressing cells, was labelled as the representative targeting moiety onto the cell surface by BCC. The surface‐edited EVs effectively accumulated into the target tissues of the animal models with rheumatoid arthritis and tumour, primarily owing to prolonged circulation in the bloodstream and the active targeting mechanism. Overall, these results suggest that BCC combined with MGE is highly useful as a simple and safe approach for the surface modification of EVs to modulate their in vivo fate.

## 1 INTRODUCTION

Extracellular vesicles (EVs) are extracellular membrane vesicles (30–200 nm in diameter) that are endogenously released by cells and are present in most biological fluids, including blood, urine and saliva (El Andaloussi et al., 2013; Mathieu et al., 2019). Depending on the cellular origin, EVs have specific bioactive components corresponding to unique biological functions (Van Den Boorn et al., 2013). Recently, they have been identified as essential mediators in intercellular communication, transferring various biological signals between cells such as lipids, proteins, mRNAs and other noncoding RNAs (Tkach & Théry, 2016). EV‐mediated cellular communication is heavily implicated in normal physiological processes such as inflammation, homeostasis and coagulation and in the pathophysiological processes of intractable diseases such as rheumatoid arthritis (RA), atherosclerosis, stroke and cancer (Boulanger et al., 2017; Chen et al., 2018; Hergenreider et al., 2012; Jayaseelan & Arumugam, 2019). These unique characteristics of EVs, therefore, have inspired researchers to investigate their potential as diagnostic biomarkers and therapeutic nanomedicines.

Owing to their similar structure to liposomes, which are composed of biocompatible phospholipid bilayers, EVs have been considered as potential nanocarriers to deliver therapeutic agents to desired sites of action (El Andaloussi et al., 2013; Luan et al., 2017). Unfortunately, when bare EVs are systemically administered, most of them are localized in the liver and spleen, implying their removal by the reticuloendothelial system (Smyth et al., 2015; Wiklander et al., 2015). For therapeutic applications of EVs, therefore, it is important to understand their in vivo biodistribution, which determines their therapeutic effectiveness and potential toxicity in clinical applications. In addition, as the in vivo fate of nanoparticles is primarily dependent on their surface characteristics, strategies for facilitating surface modification of EVs are needed. Until now, only a few technologies have been developed for the preparation of surface‐engineered EVs for in vivo applications, including physicochemical modification and genetic manipulation (Alvarez‐Erviti et al., 2011; Kooijmans et al., 2016; Smyth et al., 2014). However, there are specific cells available for genetic manipulation strategies, which are often limited by low transfection efficiency and time‐consuming processes. In addition, covalent or noncovalent membrane associations might impair the function of EV by altering the active sites of surface proteins or the flexibility of the membrane (Armstrong et al., 2017). To obtain a versatile natural carrier, EV surface‐engineering technologies should therefore be simple, elaborate and safe.

In recent years, bioorthogonal copper‐free click chemistry (BCC) has attracted considerable attention for its rapid, biocompatible and highly specific chemical reactions in cellular systems (Boyce & Bertozzi, 2011; Lim et al., 2019). Therefore, BCC has been used for various biomedical applications, including three‐dimensional cellular assembly, verification of metabolic pathways and spatiotemporal imaging of zebrafish (Boyce et al., 2011; Deforest et al., 2009; Laughlin et al., 2008). In particular, BCC has been investigated extensively for modification of cellular surfaces in combination with metabolic glycoengineering (MGE), by which unnatural glycans are introduced into living cells through the metabolism of specific precursors (Agatemor et al., 2019; Yoon et al., 2017). This reaction occurs in living cells, especially with artificially introduced unnatural glycans bearing functional groups such as azides, thiols or ketones (Du et al., 2009). However, there are no reports available regarding modification of EV by BCC via MGE for effective in vivo targeting of specific cells or tissues.

**FIGURE 1:**
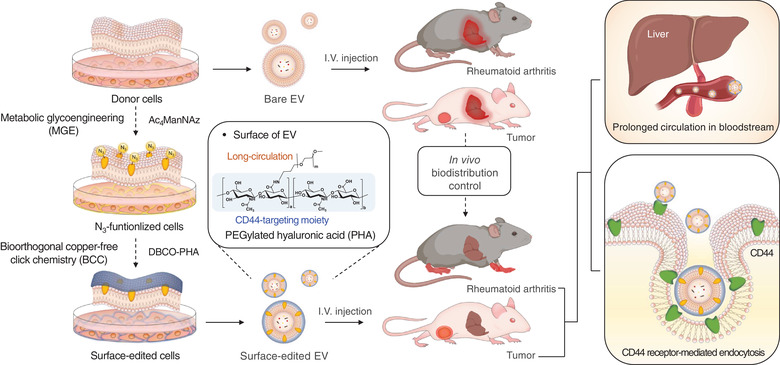
Schematic illustration of the preparation of PHA‐EVs and their in vivo fates after systemic administration. The EV surface was edited by MGE‐mediated BCC to target inflammatory diseases involving CD44

Herein, we developed an easy‐to‐adapt and highly versatile methodology to control the in vivo fate of EVs by altering their surfaces, based on BCC and MGE of EV‐secreting donor cells (Figure 1). First, unnatural sialic acids containing an azide group (‐N_3_) were exogenously introduced on the donor cells via MGE using tetraacetylated *N*‐azidoacetyl‐d‐mannosamine (Ac_4_ManNAz). Second, dibenzocyclooctyne‐terminated PEGylated hyaluronic acid (DBCO‐PHA) was prepared to specifically label the exogenously generated N_3_ group on the cells via BCC. PHA was chosen as the representative moiety for surface modification because it has shown prolonged circulation in the blood and specific binding affinity to CD44‐overexpressing tissues (Choi et al., 2011). Consequently, PHA‐decorated EVs (PHA‐EVs) were obtained by ultracentrifugation of the cell‐cultured medium without further purification. Owing to the high in vivo CD44‐targetability and stealth effects of PHA, it was hypothesized that, after their systemic administration, PHA‐EVs accumulate in CD44‐abundant tissues via their active targeting mechanism in vivo (Figure 1). The experimental conditions for surface modification were optimized in vitro, and the targeting ability of PHA‐EVs after intravenous injection was examined using a noninvasive optical imaging technique for animal models with CD44‐involving diseases such as RA and tumour.

## 2 MATERIALS AND METHODS

### 2.1 Materials

HA sodium salt (molecular weight = 10–12 kDa) was purchased from Lifecore Biomedical (MN, USA). Methoxy poly(ethylene glycol) amine (PEG‐NH_2_, molecular weight = 5 kDa) was obtained from Laysan Bio Inc. (AL, USA). Cy5.5‐DBCO and Ac_4_ManNAz were purchased from Click Chemistry Tools (AZ, USA). Thiazolyl blue tetrazolium bromide (MTT), DBCO‐amine and dimethylformamide (DMF) were obtained from Sigma‐Aldrich Co. (MO, USA). Sodium cyanoborohydride (NaBH_3_CN) was purchased from TCI (Tokyo, Japan). Poly(vinylidene fluoride) membrane was obtained from Millipore (MA, USA). Cy5.5 NHS ester dye was purchased from GE Healthcare (IL, USA). Foetal bovine serum (FBS), RPMI 1640, Dulbecco's phosphate‐buffered saline (DPBS), antibiotic‐antimycotic (AA) solution (100×) and trypsin‐trypsin‐ethylene diamine tetraacetic acid were purchased from WelGENE (Gyeongsan, Korea). NIH/3T3, CT26, RAW264.7, MDA‐MB‐231 and HCT‐116 were provided by the Korean Cell Line Bank (Seoul, Korea). Water was purified using an AquaMax‐Ultra water purification system from Younglin Co. (Anyang, Korea). All other chemicals were of analytical grade and used without further purification. Information on the antibodies used in the experiments is listed in Table S1.

### 2.2 Synthesis of DBCO‐PHA

For BCC, DBCO‐PHA was prepared by reductive amination using PHA and DBCO‐amine. First, PHA was synthesized using a previously reported procedure (Han et al., 2013). Next, PHA was chemically modified to DBCO‐PHA as follows. In brief, PHA (20 mg, 0.001 mmol) was dissolved in 4 ml of deionized water/DMF solution (1:1, v/v), to which sodium cyanoborohydride (6 mg, 0.100 mmol) and DBCO‐amine (27 mg, 0.100 mmol) were added. The resulting solution was stirred at 35°C for 120 h, sequentially dialyzed against an excess amount of methanol for 1 day and water/methanol (1:3 to 1:1, v/v) for 2 days, and then lyophilized for 3 days to obtain DBCO‐PHA. The chemical structure of DBCO‐PHA was confirmed by ^1^H NMR analysis (500 MHz; Varian Unity INOVA, CA, USA), for which the samples were dissolved in *D*_2_O and DMSO‐*d*_6_.

### 2.3 Cell viability and imaging for in vitro cell modification

To investigate the cytotoxicity of Ac_4_ManNAz by metabolic engineering, MDA‐MB‐231 (a human breast cancer cell line) and HCT‐116 (a human colon carcinoma cell line) cells were each seeded at a density of 2 × 10^3^ cells/well in 96‐well plates and stabilized for 24 h at 37°C in a humidified 5% CO_2_‐containing atmosphere. The cells were then incubated with a serial dilution of Ac_4_ManNAz (0–20 μM) for 48 h. For assessing the cytotoxicity of DBCO‐PHA by BCC after the expression of the azide group, the cells were incubated with various concentrations of DBCO‐PHA (0–10 μM) for 2 h. Afterward, cell viability was evaluated using the MTT assay, and the absorbance was measured at a wavelength of 570 nm with a microplate reader (Synergy HT Multi‐Mode Microplate Reader; Biotek, VT, USA). To visualize azide expression on the cell surface, MDA‐MB‐231 and HCT‐116 cells were each seeded at a density of 1 × 10^5^ cells onto a 35‐mm confocal dish. The cells were then incubated with various concentrations of Ac_4_ManNAz (0–20 μM) to generate azide groups on the cell surface via metabolic engineering. After 48 h, the cells were washed twice with DPBS and treated with DBCO‐Cy5.5 (10 μM) for 2 h to label the azide group by BCC.

### 2.4 Tracking of intracellular co‐localization between CD63‐GFP and PHA

To confirm the intracellular co‐localization between CD63 and PHA during EV genesis, MDA‐MB‐231 cells were transfected with pCT‐CD63‐GFP (pCMV, Exosome/Secretory, CD63 Tetraspanin tag) plasmid (System Bioscience) to construct the stable MDA‐MB‐231‐CD63‐GFP cell line (Im et al., 2019). CD63‐GFP‐transfected MDA‐MB‐231 cells were seeded in a confocal dish at a density of 1 × 10^5^ cells with RPMI 1640 medium, containing 10% FBS, 1% AA and 20 μM Ac_4_ManNAz, for 48 h. Afterward, the cells were washed twice with DPBS and treated with DBCO‐PHA‐Cy5.5 (10 μM) for 2 h. After washing twice with DPBS, the cells were incubated in serum‐free media for 24 h and fixed with 4% paraformaldehyde solution, after which the cells were observed using a confocal laser microscope (Zeiss LSM 700; Carl Zeiss, Oberkochen, Germany) in the BIORP of Korea Basic Science Institute (KBSI).

### 2.5 Preparation of PHA‐EVs

MDA‐MB‐231 and HCT‐116 cells were separately seeded in 150‐pi dishes each at a density of 5 × 10^6^ cells with RPMI 1640 medium, containing 10% FBS and 1% AA, at 37°C in a humidified 5% CO_2_‐containing atmosphere. To generate azide groups on the cell surface via metabolic engineering, the cells were incubated with 20 μM Ac_4_ManNAz for 48 h. The cells were then washed twice with DPBS (pH 7.4) and incubated with 10 μM DBCO‐PHA for an additional 2 h at 37°C. To remove the FBS‐derived EVs, the cells were washed twice with DPBS (pH 7.4) and incubated with FBS‐depleted RPMI 1640 medium (Figure S1). After 24 h, the supernatant was collected after centrifugation at 2000 × *g* for 20 min, followed by filtration using 0.22‐μm filters to remove cell debris from the medium. Sequentially, the filtered supernatant was ultracentrifuged at 120,000 × *g* for 2 h and washed twice with PBS for an additional 2 h to obtain PHA‐EVs. The pellets of PHA‐EVs were resuspended in PBS and stored at −70°C before use. For in vitro cell and in vivo animal experiments, EVs and PHA‐EVs were labelled with Cy5.5 NHS ester dye. EVs and PHA‐EVs (5 × 10^9^ each) were mixed with 100 μg of Cy5.5 NHS ester dye, and the solution was incubated overnight at 4°C. Next, the unbound dye was removed by a PD‐10 desalting column (GE Healthcare, IL, USA).

### 2.6 Characterization of PHA‐EVs

To verify the successful modification of PHA‐EVs, biolayer interferometry (BLI) experiments were performed on a BLItz system (ForteBio, CA, USA). First, 50 μg/ml of recombinant human CD44‐Fc chimera was immobilized on a Protein A biosensor (ForteBio). The sensors were then washed with the kinetics buffer and reacted with EVs and PHA‐EVs for the association steps. After these steps, the sensors were immersed in the kinetics buffer for the dissociation steps. As reference signals of kinetics assay, the kinetics buffer was used as an association and dissociation buffer. The morphology of PHA‐EVs was observed by transition electron microscopy (TEM) (JEM‐2100F; JEOL Ltd., Tokyo, Japan) at an accelerating voltage of 200 kV. For TEM sample preparation, EVs and PHA‐EVs were dropped on a 200‐mesh carbon‐film‐coated grid, negatively stained with 2% uranyl acetate for 1 min and washed with deionized water. The size distribution and amount of PHA‐EVs were measured using nanoparticle tracking analysis (NanoSight LM10; Malvern Instruments, Malvern, England). The parameters of nanoparticle tracking analysis measurement were set as follows: Capture duration: 30 s, shutter speed: 30 ms, screen gain: 10, detection threshold: 5 and numbers of frame: 20–40 particles/frame.

### 2.7 Cell culture

NIH/3T3 (a mouse fibroblast cell line), PC3 (a human prostate cancer cell line), CT26 (a mouse colorectal carcinoma cell line) and RAW264.7 (a mouse macrophage cell line) cells were separately seeded in 35‐mm confocal dishes each at a density of 1 × 10^5^ cells with RPMI 1640 medium containing 10% FBS and 1% AA at 37°C in a humidified 5% CO_2_‐containing atmosphere.

### 2.8 Cellular uptake

For in vitro cellular uptake studies, RAW264.7 cells were seeded in 35 mm confocal dishes with lipopolysaccharide (500 ng/ml) and interferon‐γ (20 ng/ml). After 24 h, these cells were incubated in serum‐free media containing Cy5.5‐labeled EVs or Cy5.5‐labeled PHA‐EVs (1 × 10^7^ particles/dish) for 12 h at 37°C. Afterward, the cells were washed twice with DPBS and fixed with 4% paraformaldehyde solution. After 4,6‐diamidino‐2‐phenylindole (DAPI) mounting, the cells were observed using a confocal laser microscope (TCS SP8 HyVolution; Leica Microsystems CMS GmbH, Germany) with 405 diode (405 nm), Ar (458, 488 and 514 nm) and He‐Ne (633 nm) lasers. The images were then quantified using Image J software. Briefly, after extraction of red colour from the images via split‐channel function, the fluorescent signals were calculated based on brightness values.

For competition assay, activated RAW264.7 cells were incubated in 10 mg/ml of HA for 2 h. Afterward, the Cy5.5‐labeled EVs or Cy5.5‐labeled PHA‐EVs (1 × 10^7^ particles/dish) were added to the cell medium. The cells were then washed twice with DPBS and fixed with 4% paraformaldehyde solution. After DAPI staining, the cells were observed using a confocal laser microscope.

### 2.9 Flow cytometry

EVs and PHA‐EVs (2 × 10^9^ each) were incubated with 50 μl of magnetic microbeads (6.5 μm in diameter, 6000 beads) coated with CD9 or CD63 antibody (ExoStep; Immunostep, Salamanca, Spain) overnight at room temperature with gentle mixing, according to the guideline of International Society for Extracellular Vesicles (ISEV) (Welsh et al., 2020). The bead‐bound EVs were washed in buffer and placed on a magnet for 5 min before the supernatant was discarded. The bead‐bound EVs were resuspended in buffer and incubated with anti‐CD9 antibody (1 μg, 1:200) or anti‐CD63 antibody (1 μg, 1:200) for 1 h at 4°C with gentle mixing. After being washed thrice with a buffer, the antibody‐attached EVs were incubated with Alexa Fluor 488 goat anti‐rabbit IgG secondary antibody (1:200). The labelled EVs were washed with buffer and analyzed using a flow cytometer (Guava easyCyte; EMD Millipore, MA, USA).

### 2.10 Acetylcholinesterase assay

The biological activity of acetylcholinesterase (AchE) was assessed, according to the manufacturer's protocol for Amplite Colorimetric Acetylcholinesterase Assay Kit (AAT Bioquest, CA, USA). Briefly, 2.5 × 10^8^ particles of EVs or PHA‐EVs were added in 96‐well plates with 50 μl of reaction buffer and incubated in the dark room for 30 min at room temperature. Then, the absorbance was measured at a wavelength of 405 nm by a microplate reader. The enzymatic activity was calculated based on the standard curve, obtained by using AchE standard solutions (0–100 mU/ml).

### 2.11 Western blotting assay

Proteins of the MDA‐MB‐231 cells, PHA‐decorated MDA‐MB‐231 cells, EV_MDA_ and PHA‐EV_MDA_ were separated via electrophoresis using 10% sodium dodecyl sulphate polyacrylamide gel electrophoresis (SDS‐PAGE) gel. Anti‐CD9 antibody (1:500), anti‐calnexin antibody (1:1000), anti‐GM130 antibody (1:1000), anti‐TSG101 antibody (1:1000) and anti‐β‐actin antibody (1:2000) were used for primary antibodies. As a secondary antibody, the HRP‐conjugated anti‐rabbit IgG (1:1000) or HRP‐conjugated anti‐mouse IgG (1:1000) were used, and the blotted membranes were observed using a biomolecular imager (LAS‐3000; Fuji Photo Film, Japan). The molecular weights of the protein were identified with the protein ladder (Precision Plus Protein Dual Color Standards; BIO RAD, USA).

### 2.12 In vitro macrophage polarization

For in vitro cellular uptake studies, RAW264.7 cells were seeded in 35 mm confocal dishes at a density of 1 × 10^5^ cells with lipopolysaccharide (500 ng/ml) and interferon‐γ (20 ng/ml) for 12 h. The cells were then incubated with EVMDA or PHA‐EVMDA (1 × 10^8^ particles/dish) for 48 h, washed twice with DPBS and fixed with 4% paraformaldehyde solution. Afterward, the cells were incubated with 0.2% Triton‐X in DPBS for 10 min, washed twice with DPBS and blocked with 1% BSA for 45 min. The cells were treated with anti‐iNOS antibody Alexa Fluor 647 and anti‐CD206 antibody Alexa Fluor 488 at 4°C for 12 h. The resulting cells were stained with DAPI and observed under a confocal laser microscope.

### 2.13 In vivo biodistribution of PHA‐EVs

All experiments with live animals were performed in compliance with the relevant laws and institutional guidelines of Sungkyunkwan University and approved by the institutional committees. To observe the in vivo biodistribution of PHA‐EVs, two types of animal model were prepared. PC3 tumour‐bearing mice were established by injecting a suspension of 1 × 10^6^ PC3 cells into the subcutaneous tissue of nude mice (Orientbio Inc., Seongnam, Korea). Collagen‐induced arthritis (CIA) mouse models were established by the following steps. Male 6‐week‐old DBA1/J mice (Central Lab Animal Inc., Seoul, Korea) were injected intradermally at the tail with 200 μg of bovine type II collagen (2 mg/ml; Chondrex, Redmond, WA, USA) emulsified in 100 μl of complete Freund's adjuvant (4 mg/ml) (Courtenay et al., 1980). On day 21, the mice received a booster immunization of CII emulsified in incomplete Freund's adjuvant. When the tumour volume of PC3 tumour‐bearing mice reached 150–200 mm^3^ and 42 days after the primary immunization of CIA model mice, 200 μl of solution containing Cy5.5‐EVs or Cy5.5‐PHA‐EVs (1 × 10^9^ particles/head) was injected via the tail vein of each mouse (*n* = 3 per group). The time‐dependent biodistribution was observed using an IVIS Lumina III In vivo Imaging System (Caliper Life Sciences, MA, USA) with a 670‐nm pulsed laser diode. To observe the organ distribution, the major organs and tumours (or inflamed joints) were obtained and visualized using the IVIS Imaging System. All fluorescence images were obtained at wavelengths of 660 nm (excitation) and 710 nm (emission). The images were then quantified using embedded software.

### 2.14 Immunohistochemistry

For histological analysis, the dissected tumours were retrieved and fixed in a 4% (v/v) buffered formalin solution. For analysis of inflamed joints in CIA mice, the joints were decalcified for 48 h using 5% (v/v) formic acid solution and fixed in 4% (v/v) buffered formalin solution. The paraffin sections of each tissue were then sliced (5 μm in thickness). The tissue sections were immunostained with polyclonal anti‐CD44 antibody (Abcam, Cambridge, UK) and Alexa Fluor 488 goat anti‐rabbit IgG secondary antibody (Invitrogen, CA, USA), according to the manufacturer's instructions. Fluorescence images were observed using a confocal laser microscope (TCS SP8 HyVolution; Leica Microsystems CMS GmbH) with 405 diode (405 nm), Ar (458, 488 and 514 nm) and He‐Ne (633 nm) lasers.

## 3 RESULTS

### 3.1 Characterization of surface‐edited cells based on BCC and MGE

**FIGURE 2:**
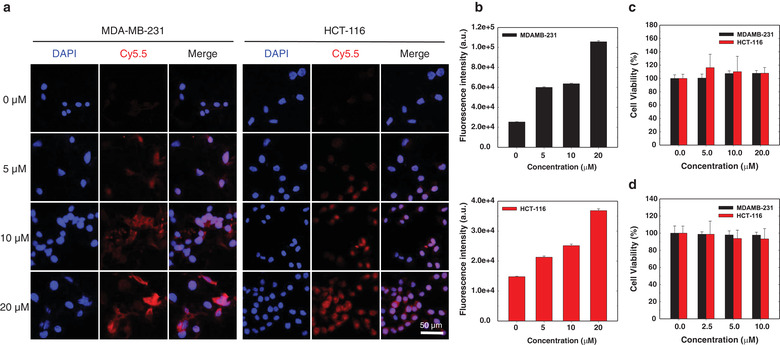
In vitro optimization of MGE‐mediated BCC for EV‐secreting donor cells. (a) Dose‐dependent expression of the azide group on MDA‐MB‐231 and HCT‐116 cells at different concentrations of Ac_4_ManNAz. (b) Fluorescence intensity of the azide group on MDA‐MB‐231 and HCT‐116 cells at different concentrations of Ac_4_ManNAz. (c) Cytotoxicity of Ac_4_ManNAz and (d) DBCO‐PHA. Error bars represent standard deviation (*n* = 5)

To obtain surface‐edited EVs, the cellular membrane needs to be properly modified using simple, safe chemistry. In this study, for cellular membrane modification, the EV‐secreting donor cells were metabolically engineered using Ac_4_ManNAz as a precursor, followed by chemical conjugation between the azide groups on the cells and the cyclic alkyne groups of DBCO‐PHA as a CD44‐targeting moiety. To introduce the in vivo CD44‐targeting moiety, PHA was chemically modified by reductive amination with DBCO‐amine. In the ^1^H NMR spectrum of DBCO‐PHA, the methyl group at the C_2_ position of *N*‐acetyl glucosamine in HA appeared at 1.8 ppm, whereas the ethylene group in PEG and the aryl group in DBCO were observed at 3.6 and 7–8 ppm, respectively (Figure S2). To identify the optimal conditions for MGE, the azide groups on the cell membrane were labelled with clickable DBCO‐Cy5.5 at various concentrations of Ac_4_ManNAz (Figure 2a). As expected, the Cy5.5 fluorescent signals in both MDA‐MB‐231 and HCT‐116 cells were enhanced by Ac_4_ManNAz in a dose‐dependent manner (Figure 2b). On the contrary, no signals were detected for the cells without Ac_4_ManNAz treatment. These data indicate that the expression level of the azide group on the cell surface is proportional to the Ac_4_ManNAz concentration for the cell treatment. To evaluate the safety of MGE and BCC to the donor cells, the viabilities of MDA‐MB‐231 and HCT‐116 cells were determined using the MTT assay at different concentrations of Ac_4_ManNAz and DBCO‐PHA (Figure 2c,d). For both cell types, there was no significant toxicity at concentrations up to 20 μM of Ac_4_ManNAz and 10 μM of DBCO‐PHA. These results imply that surface modification based on MGE is not significantly toxic to the donor cells, which is consistent with the results reported by another group (Han et al., 2018). Based on these data, PHA‐EVs for additional experiments were prepared in the presence of 20 μM Ac_4_ManNAz for MGE and 10 μM DBCO‐PHA for BCC.

### 3.2 Biogenesis of surface‐edited EVs

**FIGURE 3:**
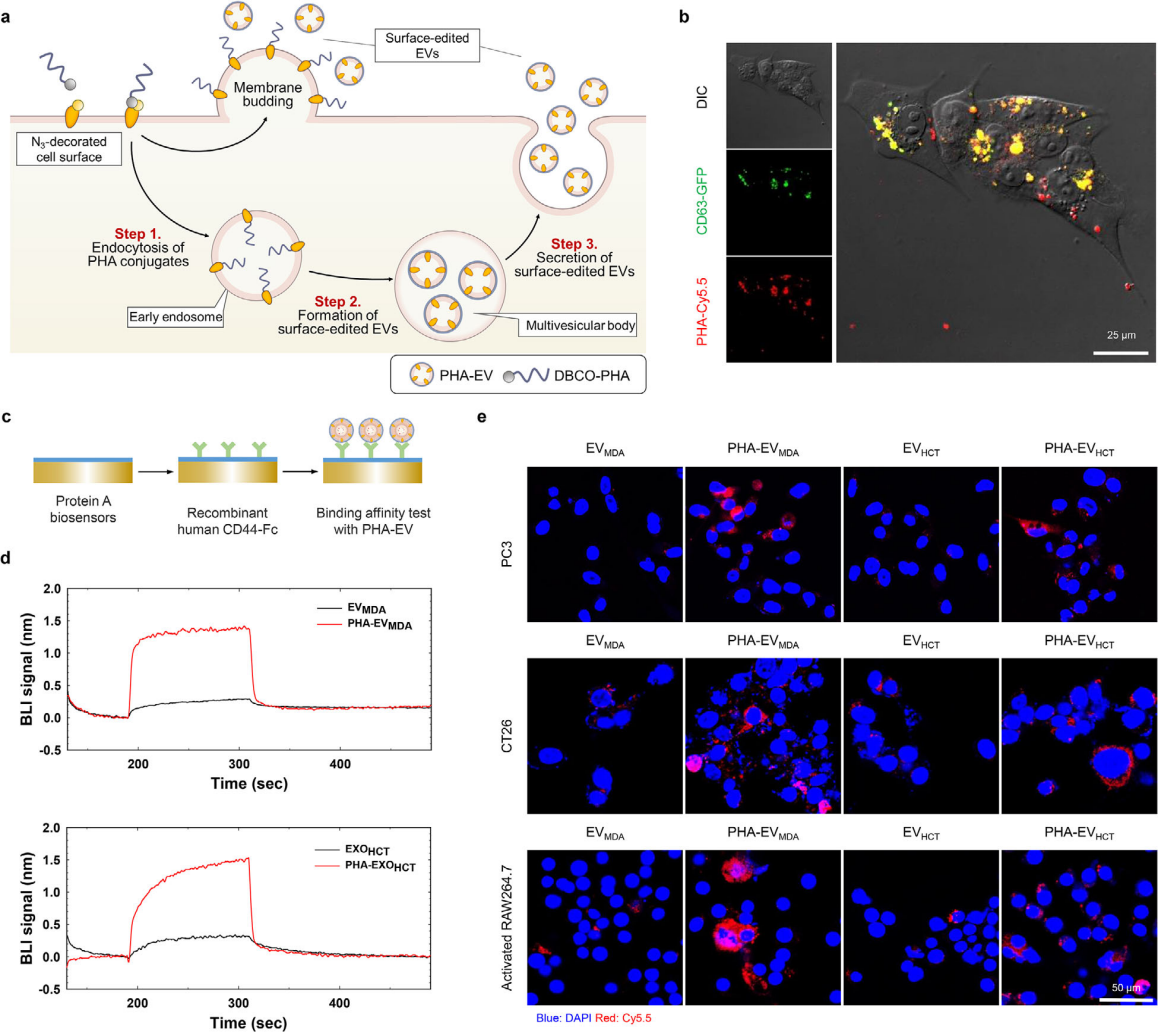
Biogenesis of surface‐edited EVs. (a) Schematic illustration of the secretion of surface‐edited EVs. (b) Confocal microscopy images of CD63‐GFP (green) and PHA (red) in MDA‐MB‐231 CD‐63 GFP cells at 24 h post‐incubation. (c) Schematic illustration of the binding affinity test of PHA‐EV with the CD44 receptor by BLI. (d) Binding kinetics of bare EVs and PHA‐EVs on CD44‐derivatized biosensors. (e) Cellular uptake behaviours of bare EVs and PHA‐EVs in various CD44‐positive cells

Various surface proteins of EVs, such as CD63 and CD9, were integrated into EVs during their formation and secretion (Figure 3a) (Mathieu et al., 2019; Théry et al., 2002). To confirm whether PHA on the surface of the donor cells is introduced onto the surface of EVs, Cy5.5‐labeled DBCO‐PHA was treated with MDA‐MB‐231 CD63‐GFP cells, and its intracellular distribution was observed using a confocal microscope. As shown in Figure 3b, at 24 h post‐incubation, PHA was co‐localized with CD63, a surface marker of EVs, implying that PHA was integrated into EVs. Next, PHA‐EVs, derived from MDA‐MB‐231 (PHA‐EV_MDA_) and HCT‐116 (PHA‐EV_HCT_), were prepared by sequentially culturing the cells with Ac_4_ManNAz for 2 days and DBCO‐PHA for 2 h, followed by isolation of EVs using differential ultracentrifugation. To verify successful PHA conjugation on the surface of EVs, the binding affinity of PHA‐EVs to CD44 was determined using BLI. Recombinant human CD44‐Fc was immobilized on a Protein A biosensor chip to observe its interaction with PHA‐EVs (Figure 3c). Compared with bare EVs, both PHA‐EVs exhibited much stronger BLI signals by specific interactions with CD44, indicating the presence of PHA on the surfaces of EVs (Figure 3d). The in vitro cellular uptake behaviours of bare EVs and PHA‐EVs were observed using confocal microscopy after they were treated with CD44‐positive cells (PC3, CT26 and activated RAW264.7 cells). As shown in Figure 3e, fluorescence signals in the cytosols of PHA‐EV‐treated cells were much stronger than those in bare EVs. Quantitatively, the fluorescence intensities from PHA‐EVs were much higher than those from bare EVs (Figures S3–S5). Of note, the fluorescence intensity of PHA‐EV_MDA_ was much higher than that of PHA‐EV_HCT_ in CD44‐positive cells, which might be due to the donor cell‐dependent metabolism (Pan et al., 2012). As expected, no fluorescence signals were observed in CD44‐negative cells treated with bare EVs and PHA‐EVs. In addition, internalization of PHA‐EVs into the CD44‐positive cells were remarkably inhibited by the free HA (Figure S6). Overall, it was evident that PHA‐EVs were taken up by the cells via CD44 receptor‐mediated endocytosis.

### 3.3 Characterization of surface‐edited EVs

**FIGURE 4:**
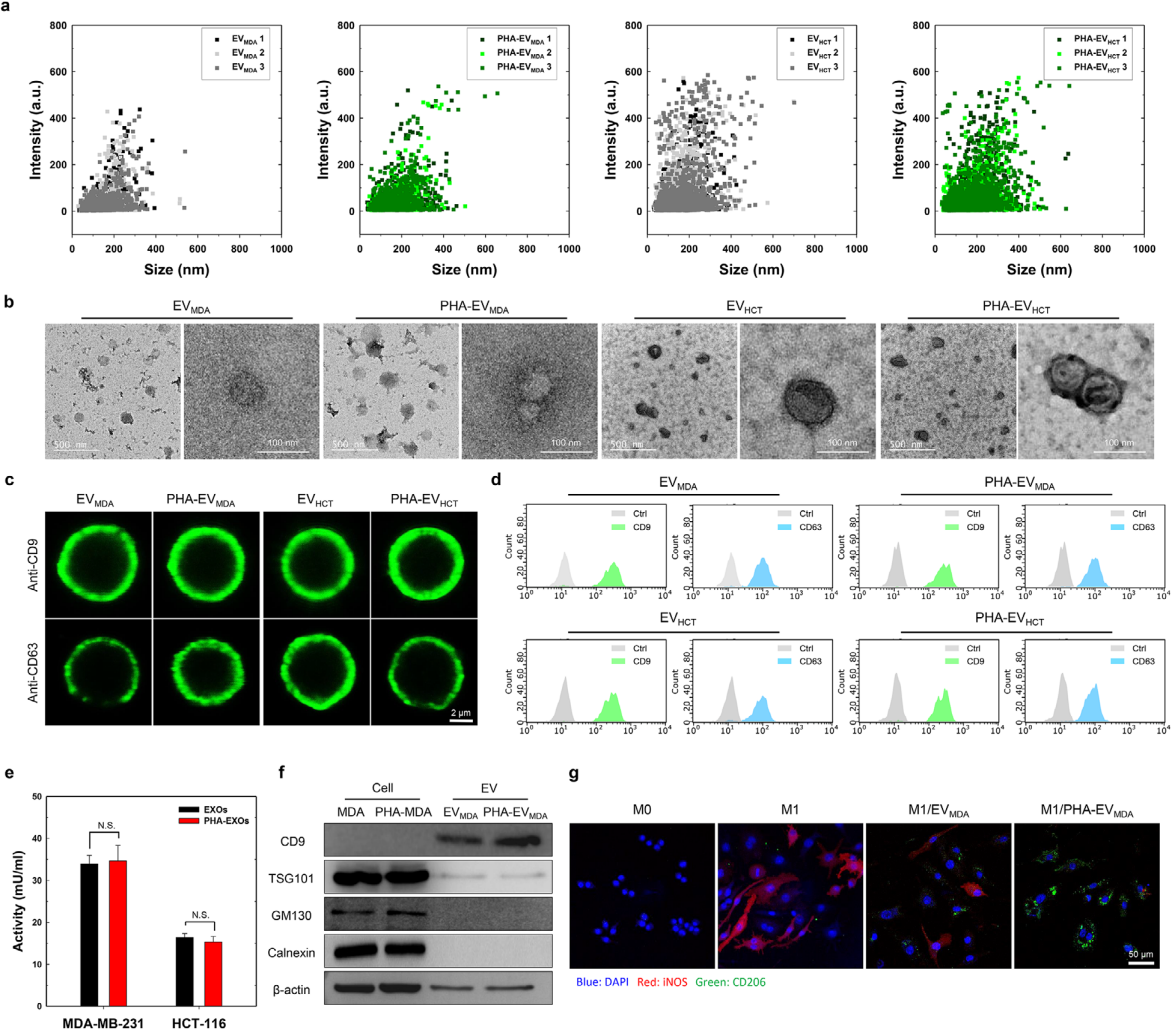
Physicochemical characteristics of PHA‐EVs. (a) Size distribution of bare EVs and PHA‐EVs (*n* = 3). (b) TEM images of bare EVs and PHA‐EVs. (c) Confocal microscopy images of surface markers on bare EVs and PHA‐EVs. (d) Flow cytometry analysis of surface markers on bare EVs and PHA‐EVs. (e) AchE activity of bare EVs and PHA‐EVs (*n* = 3). (f) Western blotting analysis of bare EV‐secreting cells, PHA‐EV‐secreting cells, bare EVs and PHA‐EVs. (g) M1–M2 macrophage polarization by the PHA‐EVs. Confocal microscopy images show iNOS (red) and CD206 (green) in cells

The physicochemical characteristics of PHA‐EVs before and after surface editing by MGE and BCC are shown in Figure 4. The particle size distribution, as shown in Figure 4a, indicated that PHA labelling on EVs slightly increased their sizes (EV_MDA_ = 166.5 ± 13.5 nm, PHA‐EV_MDA_ = 183.5 ± 8.3 nm, EV_HCT_ = 159.2 ± 7.5 nm, PHA‐EV_HCT_ = 172.5 ± 13.1 nm). According to morphological analysis by TEM, all the EVs were spherical in shape (Figure 4b). Next, to verify whether the surface editing process would affect the protein expression of EVs, PHA‐EV_MDA_ and PHA‐EV_HCT_ were characterized by the magnetic microbead‐based immune‐capturing technique (Campos‐Silva et al., 2019). To observe surface proteins of EVs, bare EVs and PHA‐EVs were reacted with magnetic beads labelled with anti‐CD9 and anti‐CD63 antibodies (Figure 4c). In microscopic images, both bare EVs and PHA‐EVs showed comparable fluorescent signals for CD9 and CD63, indicating the presence of markers of EVs on surface‐edited EVs. The markers of EVs on bare and surface‐edited EVs were also confirmed by flow cytometry analysis (Figure 4d). The expression levels of CD9 and CD63 on surface‐edited EVs were not significantly different from those on bare EVs. To confirm whether the surface‐editing technique affected the enzymatic activity of EVs, the AchE assay was conducted for bare EVs and surface‐edited EVs (Figure 4e). There were no significant differences in enzymatic activities between bare EVs and surface‐edited EVs. As shown in Figure 4f and Figure S7, western blotting analysis indicated that enriched protein markers of EVs, including CD9 and TSG101, were clearly observed on both bare EVs and surface‐edited EVs, whereas depleted protein markers of EVs, such as GM130 and Calnexin, were not detected on both EVs. Interestingly, the expression levels of CD9 and TSG101 on surface‐edited EVs were comparable to those on bare EVs. To confirm whether the MGE‐mediated BCC of donor cells affected the biological function of EVs, the effects of the EVs on M1–M2 macrophage polarization were evaluated since the ability to promote M2 polarization of macrophages has considered one of the representative biological functions of cancer cell‐derived EVs (Ham et al., 2018). As shown in Figure 4g, the in vitro immunofluorescence staining images of bare EV‐ and PHA‐EV‐treated activated RAW264.7 cells (LPS+, IFN‐γ+) were observed using confocal microscopy. Interestingly, the PHA‐EV‐treated cells had much lower iNOS fluorescence signals (red) and much higher CD206 fluorescence signals (green) than the bare EV‐treated cells (Figure S8), which might be due to the enhanced cellular uptake of PHA‐EVs by the receptor‐mediated endocytosis Figure S6). Overall, the surface‐editing technique of BCC via MGE might not affect the biological function and the expression of surface proteins of EVs.

### 3.4 CD44‐mediated targeting of inflammatory diseases

**FIGURE 5:**
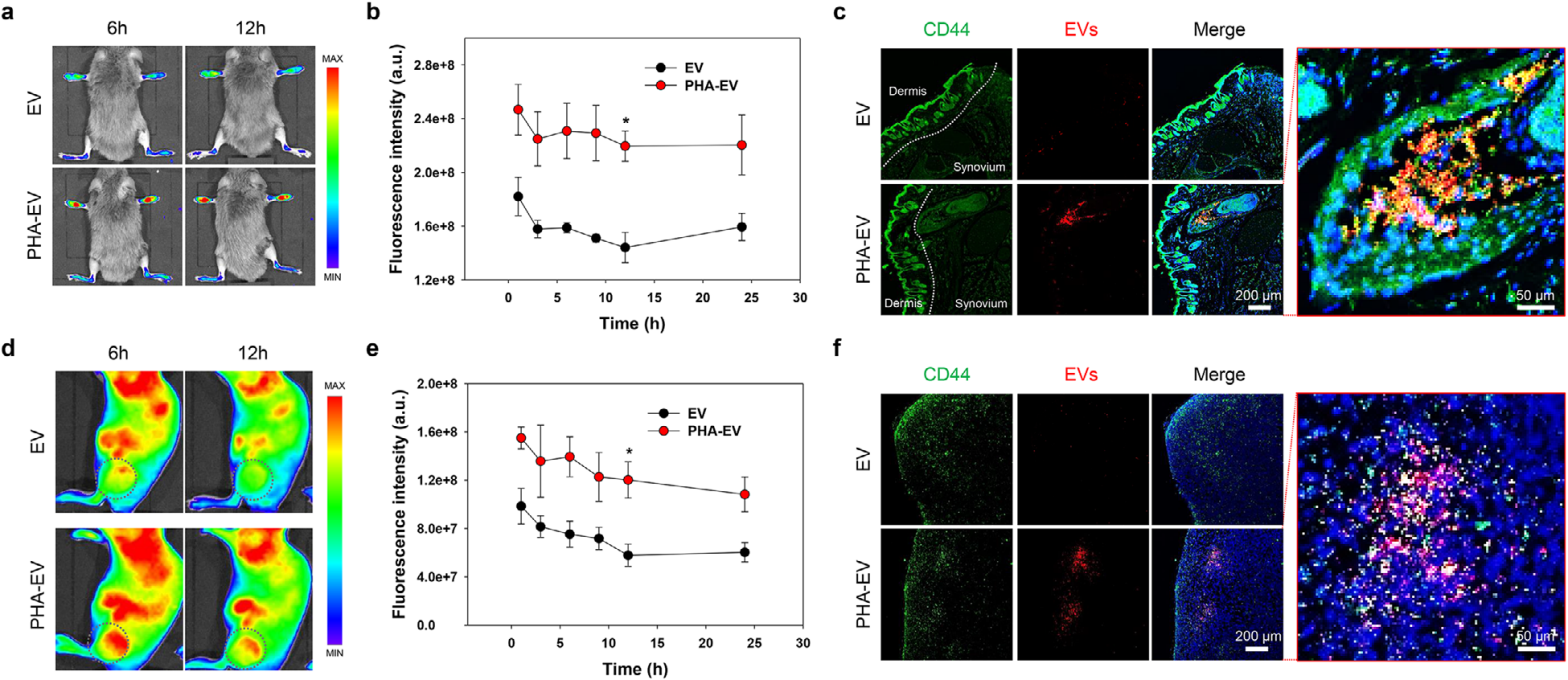
In vivo biodistribution of PHA‐EVs in inflammatory animal models with tumour and RA. (a) In vivo fluorescence images of Cy5.5‐EVs and Cy5.5‐PHA‐EVs in CIA mice. (b) Fluorescence intensity of Cy5.5‐EVs and Cy5.5‐PHA‐EVs in the inflamed joints of CIA mice as a function of time (*n* = 3). (c) Distribution of CD44 and EV in the inflamed synovium of CIA mice. (d) Whole‐body fluorescence images of Cy5.5‐EVs and Cy5.5‐PHA‐EVs in PC3 tumour‐bearing mice. (e) Fluorescence intensity of Cy5.5‐EVs and Cy5.5‐PHA‐EVs in the tumour site as a function of time (*n* = 3). (f) Distribution of CD44 and EV in tumour tissue. (b and e) *P < 0.05, analyzed by two‐way ANOVA. Error bars represent standard error

To maximize the therapeutic efficacy of EVs, it is highly important to control their in vivo fate after systemic administration. Therefore, we investigated the effects of surface editing on the in vivo biodistribution of EVs after intravenous injection into CIA mice and tumour‐bearing mice (Figure 5). PHA‐EV_MDA_ was chosen as a representative of surface‐edited EVs because it showed the highest cellular uptake behaviour to CD44‐positive cells (Figure 3e). As shown in Figure 5a, the in vivo distribution and RA targeting behaviour of PHA‐EVs were investigated using an optical imaging technique after their systemic administration into CIA mice. Interestingly, a strong fluorescence intensity of PHA‐EVs was detected at the inflamed joints and persisted with very little decrease for up to 24 h (Figure 5b). Quantitatively, the fluorescence intensities from PHA‐EV‐treated joints were 1.52‐fold higher than those from bare EVs at 12 h post‐injection. At 24 h post‐injection, the ex vivo images of the major organs and inflamed joints suggested that the fluorescent signal of PHA‐EVs at the inflamed joint was 1.8‐fold higher than that of bare EVs (Figure S9). To further evaluate the involvement of CD44 in PHA‐EV uptake, we observed the tissue distribution of CD44 and PHA‐EVs by immunohistochemistry (Figure 5c). As expected, rare fluorescent signals were observed in the inflamed synovium treated with bare EVs. In contrast, significant PHA‐EV fluorescence was detected in the inflamed synovium, and this fluorescent signal was also co‐localized with that of CD44. These results imply that PHA‐EVs can effectively target the CD44‐positive cells in the inflamed joints via the receptor‐mediated active targeting mechanism. Next, the time‐dependent whole‐body biodistribution of PHA‐EVs was evaluated using an optical imaging technique after their systemic administration into PC3 tumour‐bearing mice (Figure 5d). Interestingly, compared with bare EVs, PHA‐EVs showed enhanced fluorescence intensity at the tumour tissue, which persisted for up to 24 h (Figure 5e). Quantitatively, the fluorescence intensity of PHA‐EVs was twice as high as that of bare EVs at 12 h post‐injection. Moreover, the whole‐body fluorescence intensity of PHA‐EVs was 1.6‐fold higher than that of bare EV at 24 h post‐injection, indicating the prolonged circulation of PHA‐EVs in the bloodstream (Figure S10a, b). The ex vivo images of the major organs and tumour harvested at 24 h post‐injection suggest that the amount of PHA‐EVs at the tumour was 1.7‐fold higher than that of bare EVs (Figure S10c,d). We then examined the distribution of CD44 and PHA‐EVs in the tumour by immunohistochemistry (Figure 5f). As expected, the fluorescence signal of PHA‐EV‐treated tumour tissue was much stronger than that of bare EVs. Interestingly, the co‐localized fluorescence signals of CD44 and EVs were only detected in PHA‐EV‐treated tumour tissue. The enhanced tumour targetability of surface‐edited EVs might be due to prolonged circulation in the bloodstream, followed by the active targeting mechanism via receptor‐mediated endocytosis.

## 4 DISCUSSION

Although EVs have gained significant attention as therapeutic nanomedicines, their targeted delivery remains a challenge. The in vivo distribution of EVs is primarily determined by their surface characteristics, which can be controlled by editing strategies based on either genetic engineering or chemical modification. The former strategy, however, is only available for modifying the surfaces of EVs with genetically encodable peptides and proteins. Although the latter has been used for a wide range of molecules by harnessing noncovalent or covalent interactions, the in site‐specific modification and complicated purification steps necessary to remove the unreacted chemicals remain challenging. It should be emphasized that there are no literature available for surface‐editing methodology, allowing for highly site‐specific modification without an additional purification process.

To address the aforementioned issues in the conventional surface‐editing techniques of EVs, we developed an easy‐to‐adapt and novel methodology based on BCC and MGE of EV‐secreting donor cells (Figure 1). In this study, PHA‐EVs were prepared as model surface‐edited EVs by a highly site‐specific manner without an additional purification process. It is noteworthy that there has been no report on the methodology for isolating surface‐edited EVs by directly attaching the targeting moiety to the surface of cells. As shown in Figure 3b, PHA was incorporated into the early endosomes and co‐localized with CD63 in MDA‐MB‐231 CD63‐GFP cells. These results imply that PHA as a CD44‐targeting moiety is effectively involved in the formation process of EVs (Figure 3a).

Of note, there were no significant cytotoxic effects of DBCO‐PHA and Ac_4_ManNAz, which were used for BCC and MGE of EV‐secreting donor cells (Figure 2c,d). Moreover, our previous study showed that low‐dose Ac_4_ManNAz did not affect the extracellular acidification and oxygen consumption rates of stem cells (Lee et al., 2017). Thus, the surface‐editing technique based on BCC and MGE might not affect the biological function of EV‐secreting donor cells. In the comparison study of physicochemical characteristics and surface protein expression levels between bare EVs and PHA‐EVs, there were no significant differences in the structure of EVs and the expression levels of CD9 and CD63. This suggests that the BCC and MGE of EV‐secreting donor cells do not affect the intrinsic properties of EV.

In summary, we developed a facile, safe and elaborate methodology to endow EVs with an enhanced targeting ability in vivo by BCC via MGE of EV‐secreting donor cells. This novel surface‐editing strategy can introduce targeting moieties onto EVs without negative effects on the viability of EV‐secreting cells and their protein expression. It was evident that the cell surface of donor cells could be safely, simply and specifically engineered by BCC and MGE. The PHA‐EVs prepared from the engineered cells showed preferential binding to CD44 receptors, leading to specific cellular uptake by receptor‐mediated endocytosis. In the in vivo test, the PHA‐EVs exhibited high targeting efficiency with prolonged circulation in the bloodstream of animal models with tumour and RA. Overall, it is evident that surface editing of EVs is highly useful for modulating the in vivo fates of EVs after intravenous injection, implying high potential for MGE‐mediated BCC to develop therapeutic EVs.

## AUTHOR CONTRIBUTIONS

Dong Gil You and Jae Hyung Park designed the research and conducted the data analysis. Dong Gil You, Hwa Seung Han and Jae Hyung Park wrote the manuscript. Gyeong Taek Lim performed most of the experiments. Dong Gil You, Hansang Lee, Sol Shin, Byeong Hoon Oh, E. K. Pramod Kumar, Wooram Um, Chan Ho Kim, Seungsu Han and Seungho Lim performed the experiments. Sangho Lee, Hong Yeol Yoon, Kwangmeyung Kim, Ick Chan Kwon, Dong‐Gyu Jo and Yong Woo Cho conducted the data analysis.

## CONFLICT OF INTERESTS

The authors declare the following competing interests: Y. W. Cho is the chief executive officer of ExoStemTech Inc. D.‐G. Jo and J. H. Park are stockholders of ExoStemTech Inc. The other authors declare no competing financial interests.

## Supporting information

Supporting Information
