# Supplementary material for: Bioorthogonally surface‐edited extracellular vesicles based on metabolic glycoengineering for CD44‐mediated targeting of inflammatory diseases
Source: J Extracell Vesicles. 2021 Mar 12;10(5):e12077. doi: 10.1002/jev2.12077 (PMC7953464; doi:10.1002/jev2.12077)

(Supplementary Materials for *Journal of Extracellular Vesicles*)

**Bioorthogonally surface-edited extracellular vesicles based on metabolic glycoengineering for CD44-mediated targeting of inflammatory diseases**

Gyeong Taek Lim^1,⊥^, Dong Gil You^1,⊥^, Hwa Seung Han^1^, Hansang Lee^1^, Sol Shin^2^, Byeong Hoon Oh^1^, E. K. Pramod Kumar^1^, Wooram Um^1^, Chan Ho Kim^1^, Seungsu Han^3^, Sangho Lee^3^, Seungho Lim^4^, Hong Yeol Yoon^4^, Kwangmeyung Kim^4^, Ick Chan Kwon^4^, Dong-Gyu Jo^5,6^, Yong Woo Cho^6,7^, and Jae Hyung Park^1,2,6,*^

^1^ School of Chemical Engineering, Sungkyunkwan University, Suwon 16419, Republic of Korea

^2^ Department of Health Sciences and Technology, SAIHST, Sungkyunkwan University, Suwon 16419, Republic of Korea

^3^ Department of Biological Sciences, Sungkyunkwan University, Suwon 16419, Republic of Korea

^4^ Center for Theragnosis, Biomedical Research Institute, Korea Institute of Science and Technology, Seoul 02792, Republic of Korea

^5^ School of Pharmacy, Sungkyunkwan University, Suwon 16419, Republic of Korea

^6^ ExoStemTech Inc., Ansan 15588, Republic of Korea

^7^ Department of Chemical Engineering, Hanyang University, Ansan 15588, Republic of Korea

^⊥^ These authors contributed equally to this paper.

**Corresponding author:* ***Jae Hyung Park*** *School of Chemical Engineering, Sungkyunkwan University, 2066 Seobu-ro, Jangan-gu, Suwon 16419, Republic of Korea*

E-mail: [jhpark1@skku.edu](mailto:jhpark1@skku.edu)*
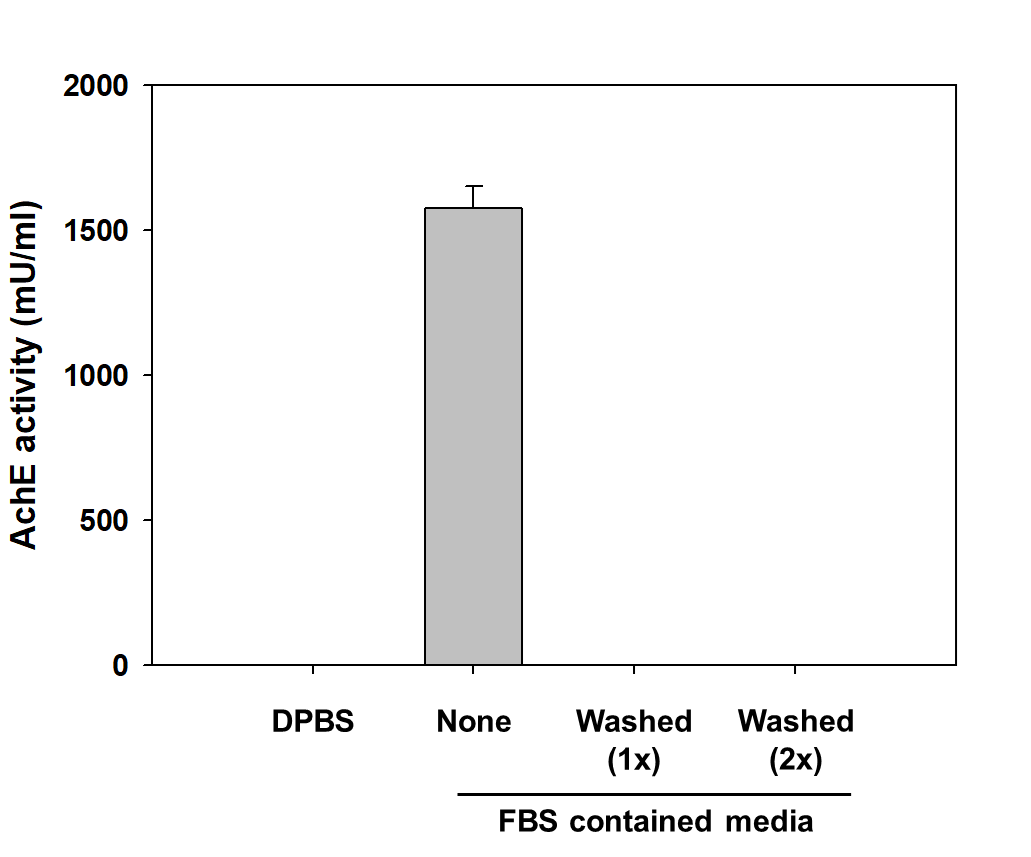
*

**Figure S1.** AchE activity in the FBS-depeletion process (*n* = 3).

**
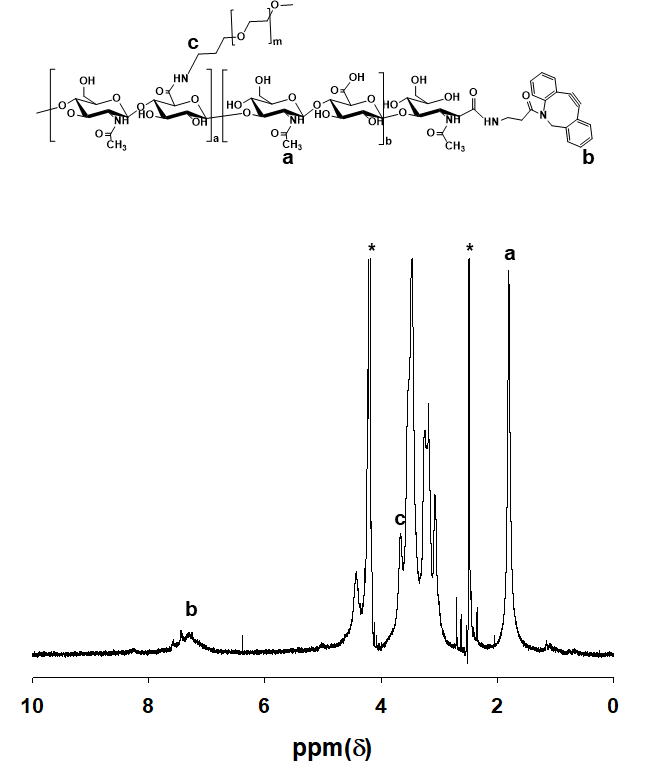
Figure S2.** ^1^H NMR spectra of DBCO-PHA in D_2_O and DMSO-d_6_.


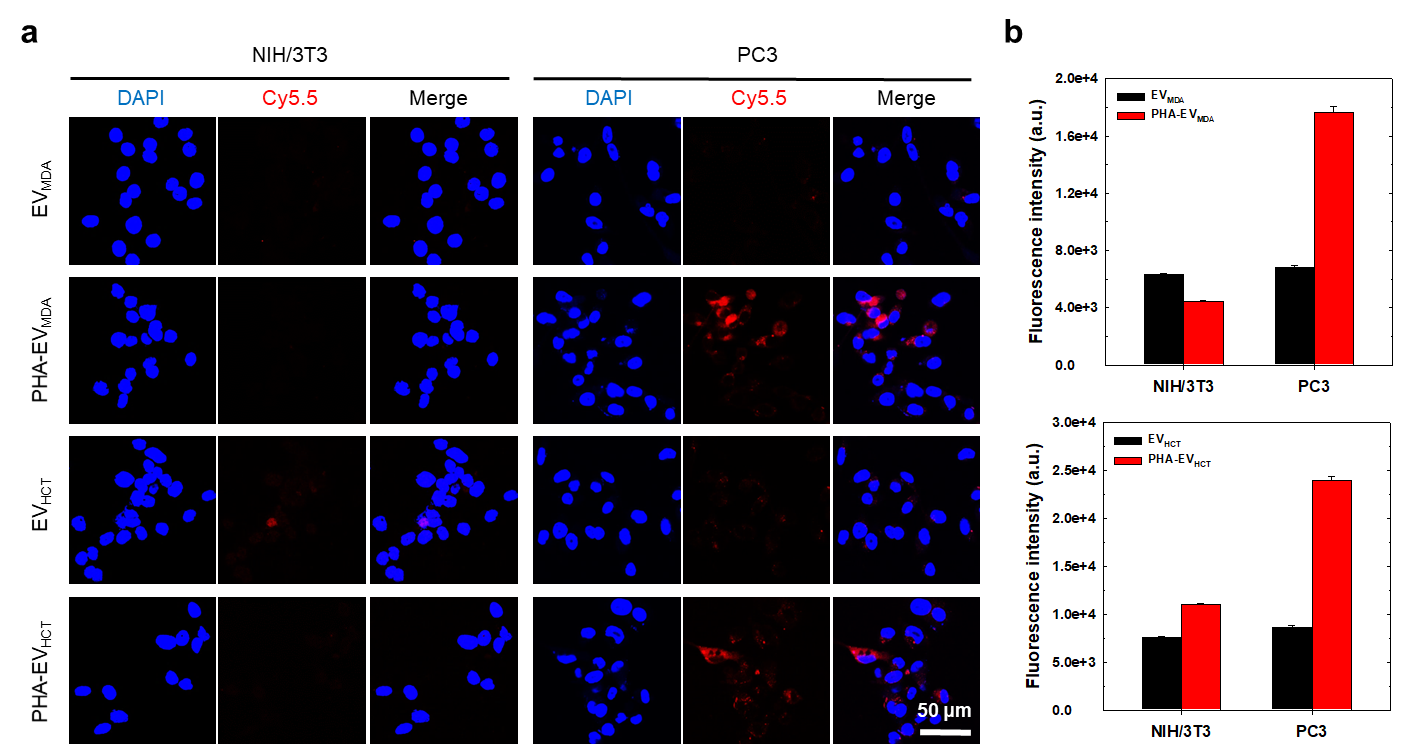


**Figure S3.** (a) Cellular uptake behaviors of bare EVs and PHA-EVs in NIH/3T3 and PC3 cells. (b) Fluorescence intensity of bare EVs and PHA-EVs in NIH/3T3 and PC3 cells.


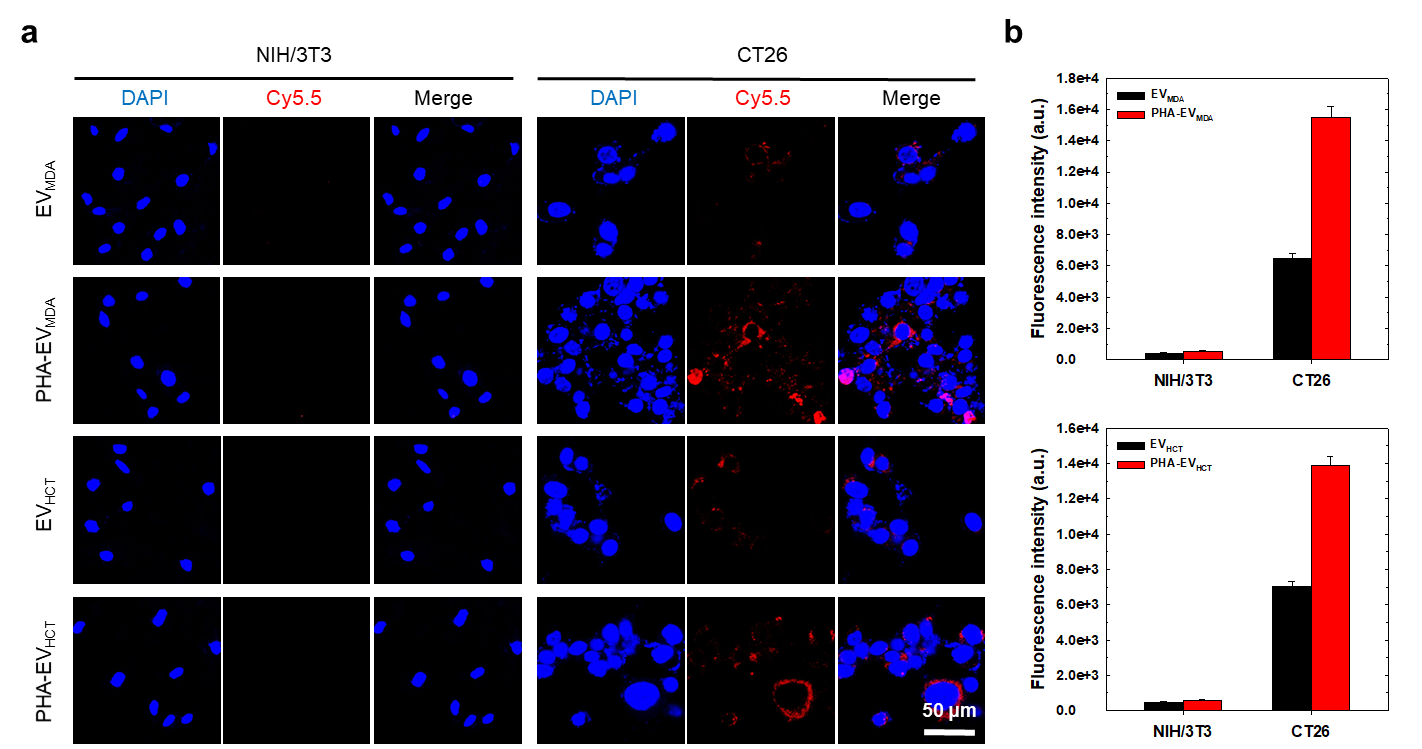


**Figure S4.** (a) Cellular uptake behaviors of bare EVs and PHA-EVs in NIH/3T3 and CT26 cells. (b) Fluorescence intensity of bare EVs and PHA-EVs in NIH/3T3 and CT26 cells.


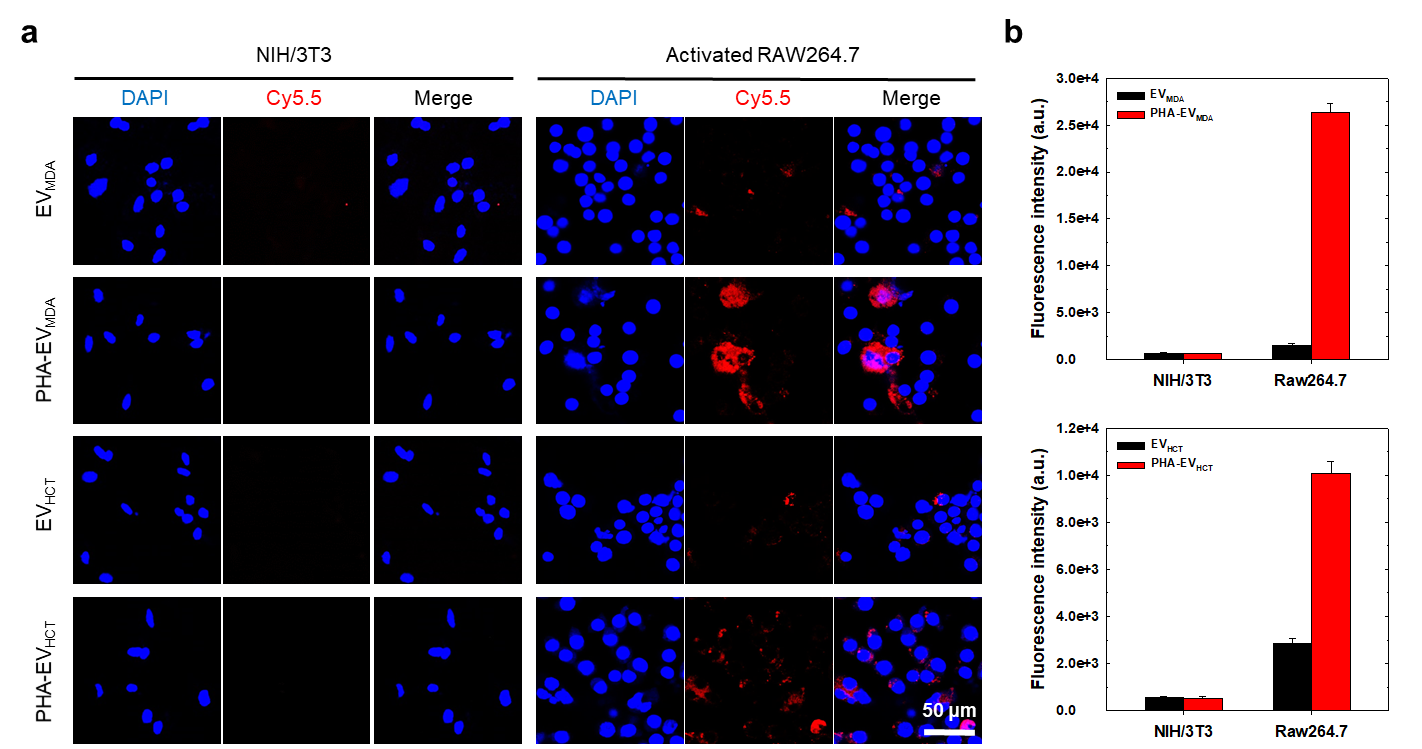


**Figure S5.** (a) Cellular uptake behaviors of bare EVs and PHA-EVs in NIH/3T3 and activated RAW264.7 cells. (b) Fluorescence intensity of bare EVs and PHA-EVs in NIH/3T3 and activated RAW264.7 cells.
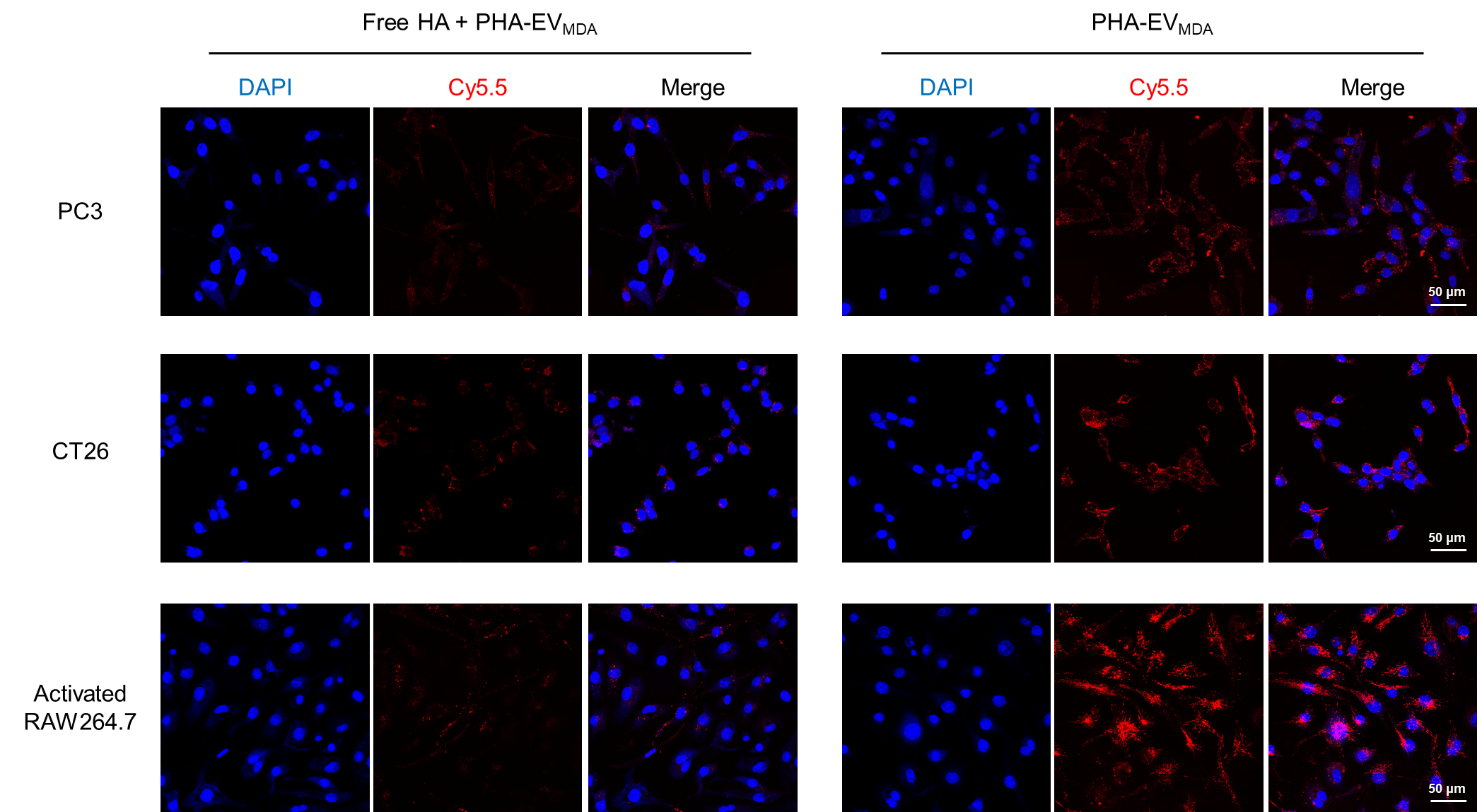


Figure S6. Confocal microscopy images for CD44-mediated endocytosis of PHA-EV_MDA_ in free HA-treated activated RAW264.7.


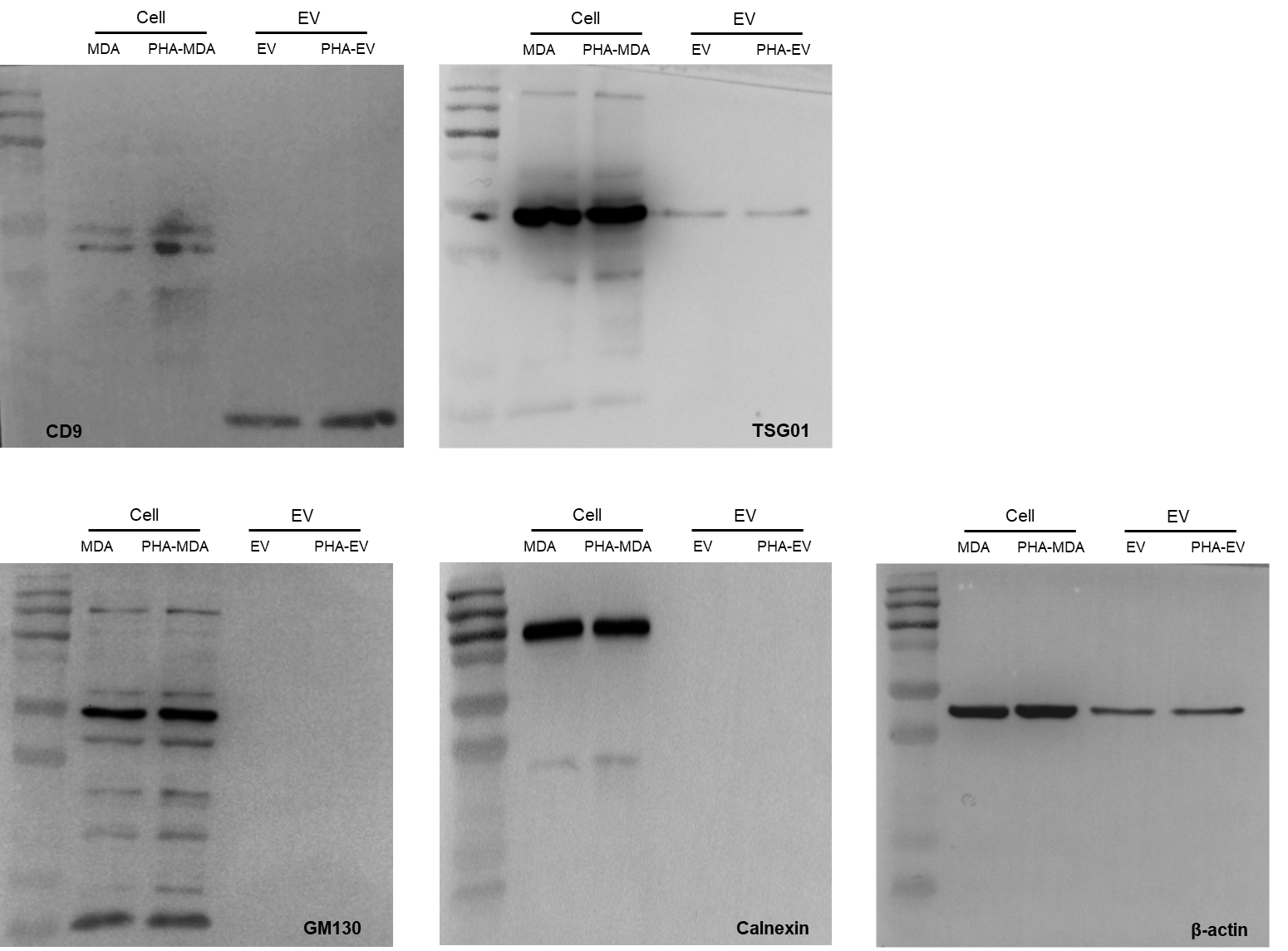


**Figure S7.** Full gel images in western blotting analysis of bare EV-secreting cells, EVs, and PHA-EVs**.**

**
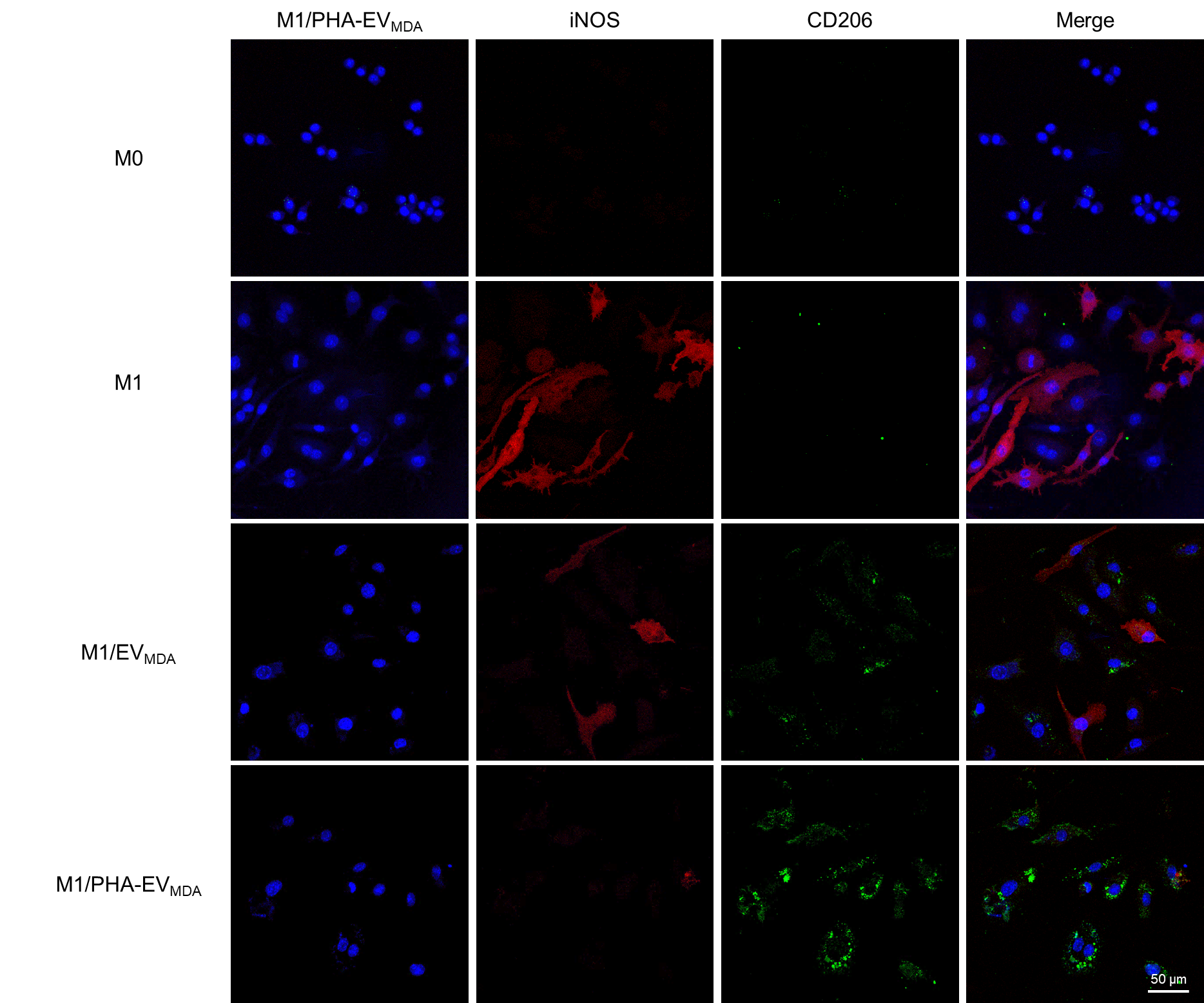
**

**Figure S8.** M1–M2 macrophage polarization by the PHA-EVs. Confocal microscopy images show iNOS (red) and CD206 (green) in cells.
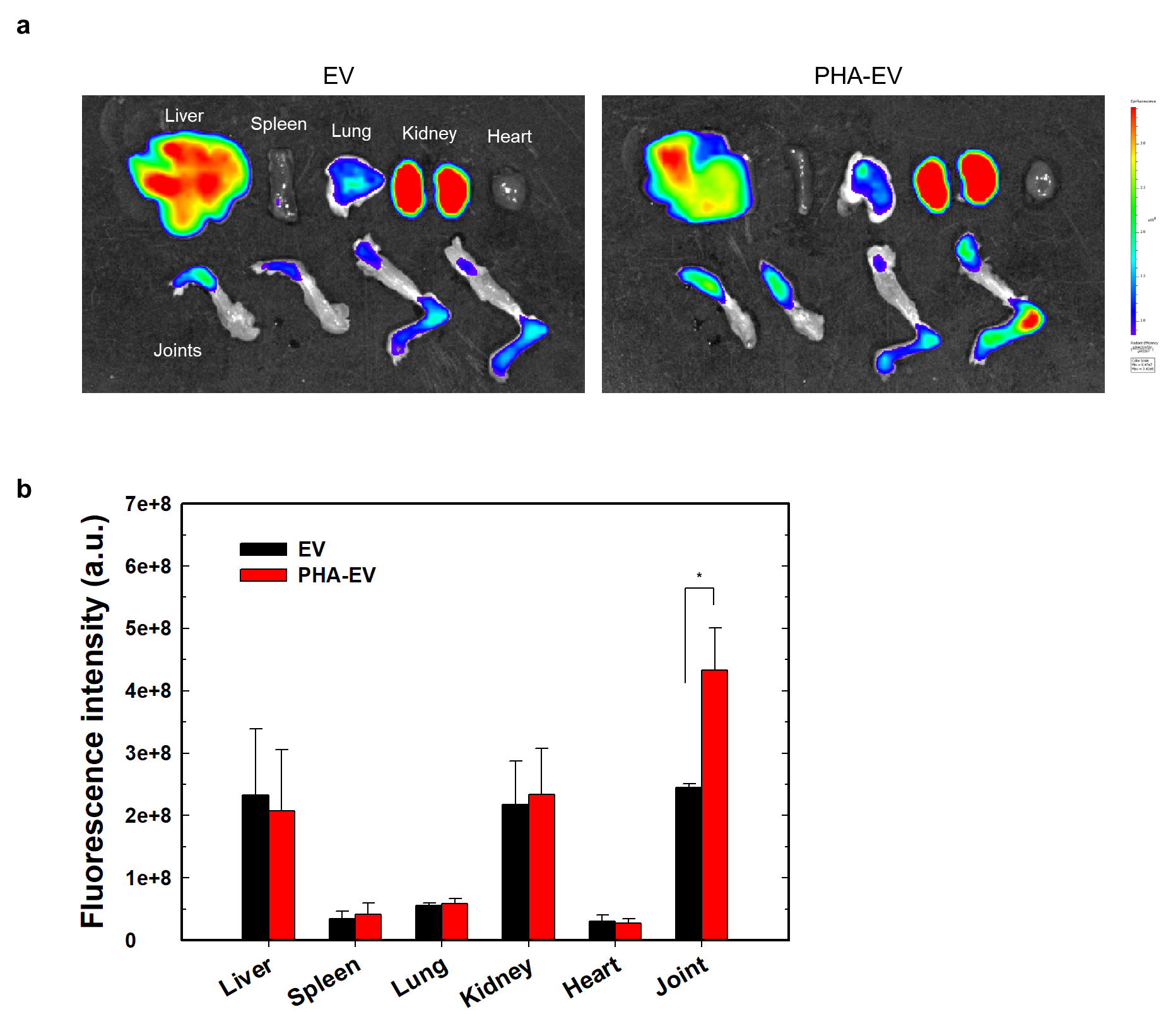


**Figure S9.** (a) *Ex vivo* organ distribution images of Cy5.5-EVs and Cy5.5-PHA-EVs at 24 h post-injection. (b) Quantification of fluorescence intensity in the inflamed joint and major organs of CIA mice (*n* = 4). **p* < 0.05, analyzed by t-test.


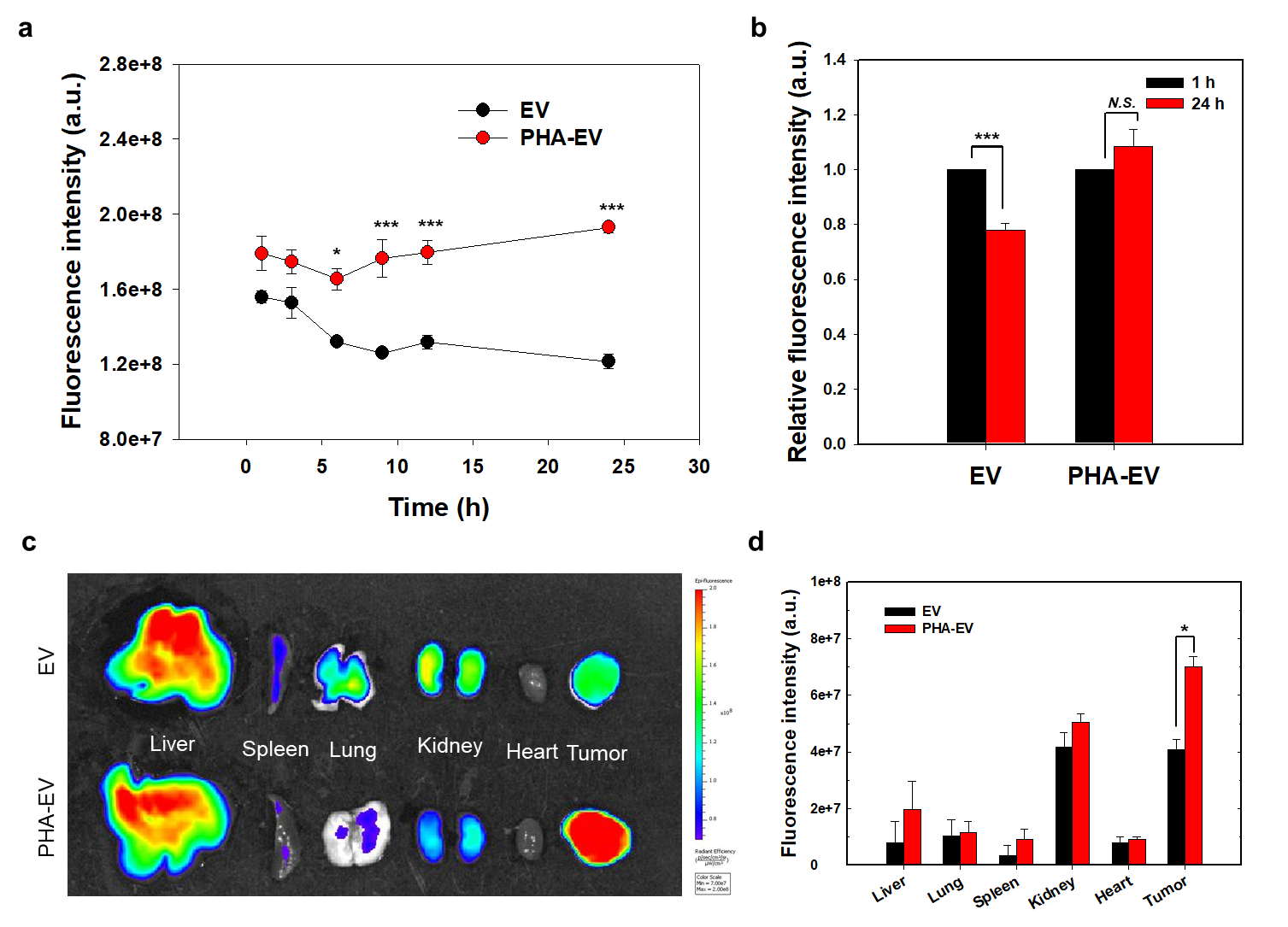


**Figure S10.** (a) Whole body fluorescence intensity of Cy5.5-EVs and Cy5.5-PHA-EVs as a function of time (*n* = 3). (b) Relative fluorescence intensity of Cy5.5-EVs and Cy5.5-PHA-EVs in the whole body at 24 h (*n* = 3). (c) *Ex vivo* organ distribution images of Cy5.5-EVs and Cy5.5-PHA-EVs at 24 h post-injection. (d) Quantification of fluorescence intensity in the tumor and major organs of PC3 tumor-bearing mice (*n* = 3). **p* < 0.01, ***p* < 0.005, and, ****p* < 0.001, analyzed by two-way (a and d) or one-way (b) ANOVA.

**Table S1.** Information on the antibodies used.


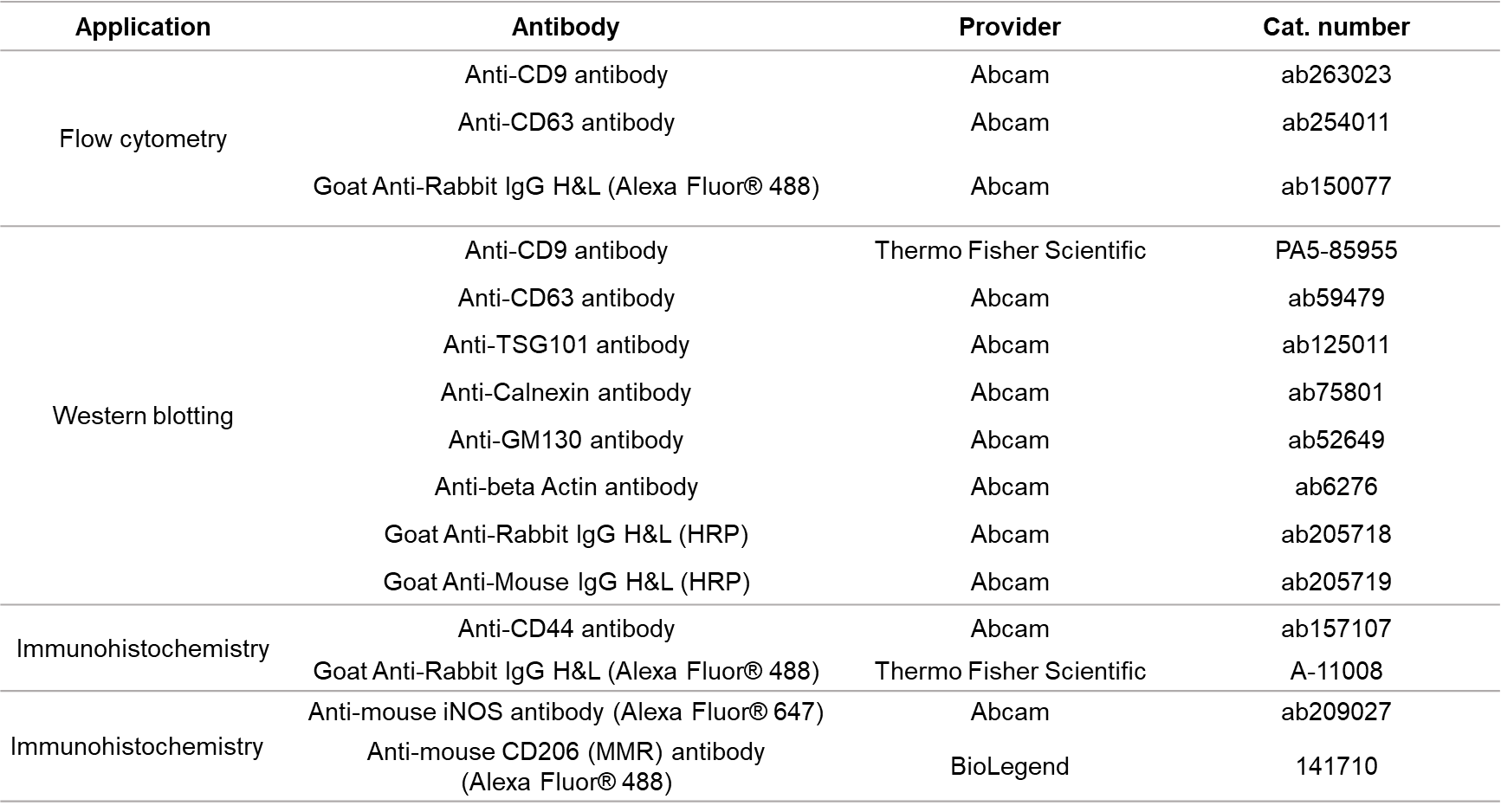

Supplement: Supplementary file 1 — Supporting Information [file JEV2-10-e12077-s001.docx]
